# Acute Gastric Dilatation: A Transient Cause of Hepatic Portal Venous Gas—Case Report and Review of the Literature

**DOI:** 10.1155/2013/723160

**Published:** 2013-05-30

**Authors:** Satya B. Allaparthi, Curuchi P. Anand

**Affiliations:** ^1^Department of Medicine, Saint Vincent Hospital, 123 Summer Street, Worcester, MA 01608, USA; ^2^Division of Gastroenterology, Department of Medicine, Saint Vincent Hospital, 123 Summer Street, Worcester, MA 01608, USA

## Abstract

Gastric pneumatosis (GP) and hepatic portal venous gas (HPVG) have typically been thought of as an ominous radiological sign associated with a grave prognosis, and the observation of HPVG on plain abdominal radiography, ultrasonography, or computed tomography is viewed as a significant finding. It is often associated with severe or potentially lethal conditions warranting urgent diagnosis and possible surgical intervention. Early studies of HPVG based on plain abdominal radiography found an associated mortality rate of 75% primarily due to ischemic bowel. However, modern abdominal computed tomography (CT) has resulted in the detection of HPVG in an increased proportion of nonfatal and benign conditions. We report a nonfatal case of HPVG in a patient with Noonan's syndrome due to acute gastric dilatation in the setting of gastric outlet obstruction caused by a congenital band that is extremely rare in adults.

## 1. Introduction

Hepatic portal venous gas (HPVG) was first described more than 55 years ago by Wolfe and Evans and was presented as a radiologic sign associated with more than 75% of mortality rate in infants with necrotizing enterocolitis (NEC) [1, 2]. Since that time, HPVG has been associated with numerous other underlying abdominal diseases, ranging from benign causes to potentially lethal diseases requiring prompt surgical intervention. Moreover, HPVG is recognized not as a specific disease entity but rather as a diagnostic clue in patients with acute abdominal pathology. Although early reports of HPVG estimated a mortality rate of 75% to 80%, more recent studies suggest mortality rates of 25% to 35% [3–5]. The observed reduction in mortality may be attributed to the increased availability of more sensitive diagnostic imaging modalities (i.e., ultrasonography (US) and computed tomography (CT)) that can detect even minute quantities of air in the portal system. In addition, there is also an increase in the proportion of nonfatal conditions reported with HPVG without associated mesenteric ischemia [6]. We report a case of HPVG in a patient with Noonan's syndrome due to acute gastric dilatation in the setting of gastric outlet obstruction caused by a congenital band that is extremely rare in adults. This study reviews the clinical data in adults from the literature and discusses the management of underlying disease.

## 2. Case Report and Management

**Figure 1:**
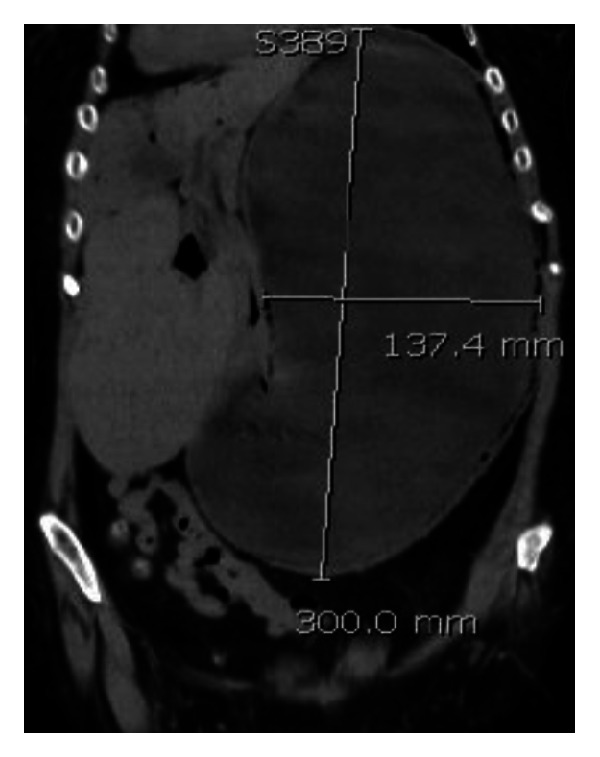
Gastric dilatation on CT scan, coronal reformat.

**Figure 2:**
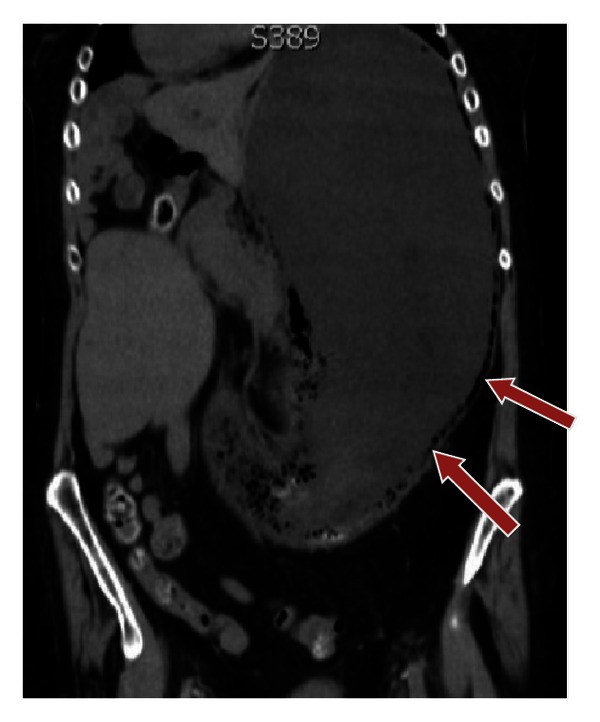
Gastric emphysema on CT scan, coronal reformat (arrows).

**Figure 3:**
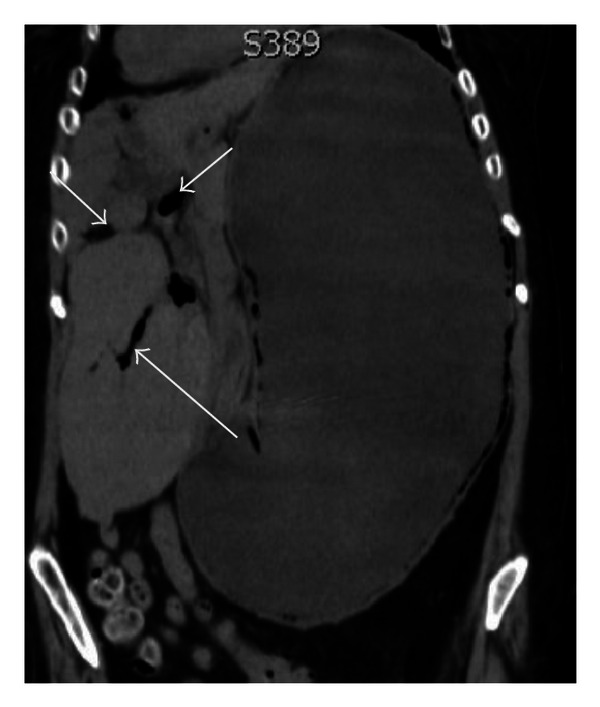
Portal venous gas on CT scan, coronal reformat (arrows).

**Figure 4:**
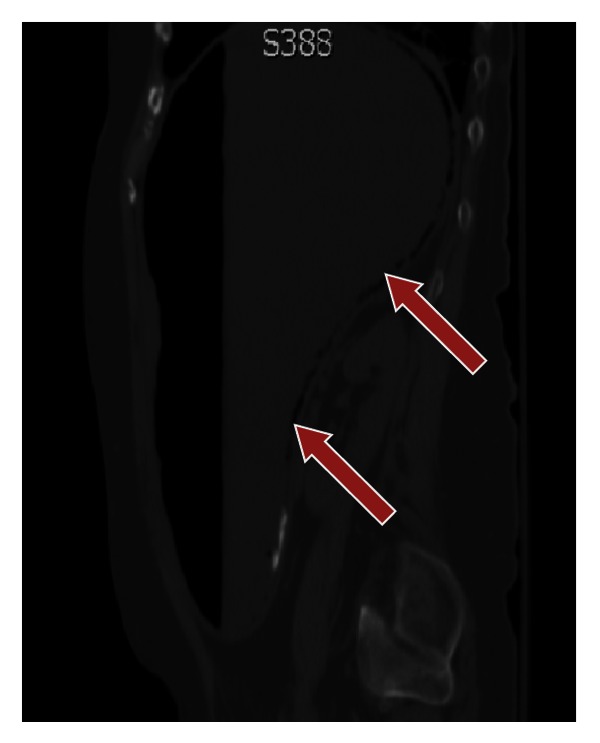
Gastric emphysema better delineated utilizing a bone/lung window on CT scan, sagittal reformat (arrows).

A 64-year-old Caucasian female with a history of Noonan's syndrome presented with sudden onset of coffee ground emesis and without any history of black tarry stool. She was recently treated for severe erosive gastritis during her last hospital admission, and an endoscopy done during that admission suggested Los Angles Class C erosive gastritis with an otherwise normal stomach and duodenum. Her past medical history is significant for a history of hypercoagulability, bilateral lower extremity deep venous thromboses, extensive bilateral pulmonary embolism, hyperlipidemia, hypertension, and dextrocardia. She complained of vague upper abdominal discomfort, and on further clinical examination, her epigastric region was soft, and distended and demonstrated no rebound. She was hemodynamically stable. Blood laboratory investigations revealed the following: white count 21.2 × 10^9^/L (3.9–11.1), hemoglobin 14.9 gm/dL, hematocrit 43.7%, platelets 322,000, sodium 145 mEq/L, potassium 2.8 mEq/L, chloride 83 mEq/L, bicarbonate 49 mEq/L, BUN 32 mg/dL, creatinine 1.90 mg/dL, glucose 188 mg/dL, calcium 11.9 mg/dL, albumin 4.3 g/dL, total bilirubin 0.3 mg/dL, alanine aminotransferase (ALT) 11 U/L, aspartate aminotransferase (AST) 27 U/L, and alkaline phosphatase of 80 U/L. The patient underwent a CT scan of the abdomen and pelvis that showed marked gastric dilatation with extensive circumferential gas (pneumatosis) and portal venous gas suspicious for gastric ischemia. The likely etiology for these findings was the presence of gastric outlet obstruction (Figures 1, 2, 3, and 4). On review of her history and comparing previous scans, there was no evidence of any prior hepatobiliary disease or prior endoscopic retrograde cholangiopancreatography. An upper gastrointestinal endoscopy showed severe mucosal congestion, submucosal hemorrhage, and bluish and purple areas with ulcers in the gastric body. Unlike the prior endoscopy, now the pylorus was visible only on retroflexion, and the scope could not be advanced into the pylorus due to looping related to the abnormal anatomy. She underwent exploratory laparotomy and was found to have one thick anomalous congenital band with blood vessels in it that was found to be the cause of gastric outlet obstruction. A patent gastrojejunostomy was created to bypass the gastric outlet obstruction, along with a feeding jejunostomy. The patient tolerated the procedure well and her symptoms completely resolved without the need for gastric resection. She was discharged home on postoperative day six.

## 3. Discussion

Since the first description of HPVG in the literature more than half a century ago, there has been no clear evidence identifying the exact nature of the gas observed in imaging studies. However, the leading hypotheses are [1] microbe-derived gas production and [2] absorbed intraluminal air [6]. In addition, the two sources of its origin have been proposed as an escape of gas from increased pressure in the bowel lumen or in an abscess with subsequent circulation into the liver, or due to the presence of gas-forming bacteria in the portal venous system and passage of gas into the circulation [7, 9, 10]. It is hypothesized that microbe-derived gases would be molecularly distinct from swallowed air. Yale et al. [11] showed that the cystic gas of pneumatosis cystoides intestinalis has been shown to represent hydrogen gas, strongly supporting a bacteriologic etiology for this gas. Further studies by Liebman et al. [7] and Kinoshita et al. [8] hypothesized that luminal air enters the capillary veins either by an impaired epithelial barrier secondary to ischemic bowel and disrupted mucosa or by increased intraluminal pressure.

Hepatic portal venous gas is recognized not as a specific disease entity but rather as a diagnostic clue in patients with underlying acute abdominal pathology. HPVG can be visualized at conventional radiography, but substantial amounts of gas must be present for detection [4]. HPVG is defined on abdominal radiography and CT as tubular branching lucencies peripherally within 2 cm of the liver capsule [12]. On the other hand, biliary gas is usually found within the central portion of the liver more than 2 cm from the liver capsule [4, 13]. In HPVG, these low-attenuation areas are caused by the accumulation of gas in the intrahepatic portal veins, from where it is carried by blood flow to the hepatic periphery. In contrast, biliary gas tends to collect centrally because of the natural movement of bile. HPVG may be diagnosed by conventional radiography, and the original HPVG literature of the 1950–60s was based on plain radiographs, primarily left lateral decubitus views [1, 2]. However, detection of HPVG on radiography is difficult and is easily overlooked [14]. US and CT have been reported to be superior to abdominal radiographs in identifying HPVG. Color Doppler US can be useful as an initial screening examination for HPVG detection. However, the utility of US is limited because of its high interoperator variability in the diagnosis of HPVG. The typical US features of HPVG are [1] echogenic particles flowing within the portal vein or [2] poorly defined, echogenic foci within the nondependent hepatic parenchyma [15–17]. Computed tomography has higher sensitivity for the detection of HPVG when compared to US and plain radiography [18]. Digital CT images also provide an opportunity to manipulate the images for ideal viewing, and many authors note that a lung window CT setting permits easy identification of both HPVG and pneumatosis intestinalis (PI), although other viewing settings may be helpful [4, 12, 19]. Increasing use of CT scan and ultrasound in the inpatient setting allows early and highly sensitive detection of HPVG in the setting of severe illnesses [20, 21], as well as recognition of an increasing number of benign and nonlife threatening causes of HPVG. The prognosis is not simply related to the presence of HPVG but is instead related to the underlying pathology [22].

Most cases of HPVG are caused by mesenteric vascular occlusion and subsequent bowel necrosis. HPVG can occur alone or in association with PI. When associated with PI, the origin of the gas seems to be intestinal ischemia. However, HPVG is not related to the severity of intestinal ischemia [7] and has even been observed with reversible ischemia. Transient cases of HPVG without clinical consequence have been observed in numerous cases of acute gastric dilatation [3, 10, 23, 24], gastric ulcer [18, 25, 26], ulcerative colitis [27], Crohn's disease [28, 29], complications of endoscopic procedures [30, 31], blunt abdominal trauma, and other isolated cases. It has been described in a number of nonsurgical conditions, including cystic fibrosis, seizures, and colchicine toxicity. Substantial literature reviews exist on iatrogenic HPVG, with HPVG observed in patients after laparoscopy [32], endoscopic retrograde cholangiopancreatography [31], liver transplantation [15], radiofrequency tumor ablation [33], and barium enema [34]. In various studies as early as 1971, higher survival rates were recognized in iatrogenic HPVG-associated illness compared with natural pathologies. Kinoshita et al. [8] reported a mortality of 39%, which is a significant reduction from 75% that was reported in 1978. The observed reduction in mortality was driven by an increase in the proportion of nonfatal conditions reported with HPVG and without associated mesenteric ischemia. In a cumulative review by Kinoshita et al. [8], there was no statistical difference in mortality rates between the operated and nonoperated cases in patients with HPVG associated with gastric dilatation, gastric ulcer, ulcerative colitis, Crohn's disease, or complications of endoscopic procedures, because these conditions are curable with conservative management alone. A review of relevant HPVG articles is presented in (Table 1).

We report a case of acute gastric dilatation that caused HPVG and gastric emphysema, which was treated by bypassing the obstruction. Our case is unique in that the diagnosis of gastric emphysema as the cause of portal venous gas occurred in the setting of acute gastric dilatation caused by a congenital band that is extremely rare in adults and related to the abnormal anatomy of this patient with Noonan's syndrome. 

## 4. Conclusion

While HPVG in previous decades was clearly defined as an ominous radiologic finding that portended a poor prognosis and a high mortality rate, today it can be a puzzling finding to reflect a benign etiology. The radiologic presence of HPVG does not necessarily indicate severe underlying pathology. It can be seen in relatively benign situations, such as following endoscopic procedures and gastric dilatation, and may require only conservative therapy. Treatment of patients with HPVG depends mainly on the underlying disease. Nowadays, with the development of advanced imaging techniques such as CT, severe pathologies such as bowel ischemia are diagnosed at much earlier stages, allowing prompt treatment and significantly reducing mortality rates. 

## Figures and Tables

**Table 1:** 

Literature source	Findings	Contribution
Wolfe and Evans [1]	A roentgenographic demonstration with postmortem anatomical correlation in a neonate with NEC.	First report of HPVG 1955.

Liebman et al. [7]	This study reviews the 64 reported cases in the literature of HPVG that appears as a branching radiolucency extending to within 2 cm of the liver capsule. Analysis of survivors indicates that the finding of HPVG requires urgent surgical exploration except when it is observed in patients with stable ulcerative colitis.	Literature review of HPVG by plain abdominal radiograph and a reported mortality of 75%.

Kinoshita et al. [8]	This study reviewed the literature on 182 cases of HPVG in adults, and the overall mortality was 39% but varied depending on the underlying disease.	Literature survey of HPVG by plain radiograph and CT

## Abbreviations

GP: Gastric pneumatosis
HPVG: Hepatic portal venous gas
NEC: Necrotizing enterocolitis
CT: Computed tomography
US: Ultrasonography.

## References

[1] Wolfe JN, Evans WA (1955). Gas in the portal veins of the liver in infants; a roentgenographic demonstration with postmortem anatomical correlation. *The American Journal of Roentgenology, Radium Therapy, and Nuclear Medicine*.

[2] Susman N, Senturia HR (1960). Gas embolization of the portal venous system. *The American Journal of Roentgenology, Radium Therapy, and Nuclear Medicine*.

[3] Benson MD (1985). Adult survival with intrahepatic portal venous gas secondary to acute gastric dilatation, with a review of portal venous gas. *Clinical Radiology*.

[4] Faberman RS, Mayo-Smith WW (1997). Outcome of 17 patients with portal venous gas detected by CT. *American Journal of Roentgenology*.

[5] Iannitti DA, Gregg SC, Mayo-Smith WW, Tomolonis RJ, Cioffi WG, Pricolo VE (2003). Portal venous gas detected by computed tomography: is surgery imperative?. *Digestive Surgery*.

[6] Nelson AL, Millington TM, Sahani D (2009). Hepatic portal venous gas: the ABCs of management. *Archives of Surgery*.

[7] Liebman PR, Patten MT, Manny J, Benfield JR, Hechtman HB (1978). Hepatic-portal venous gas in adults: etiology, pathophysiology and clinical significance. *Annals of Surgery*.

[8] Kinoshita H, Shinozaki M, Tanimura H (2001). Clinical features and management of hepatic portal venous gas: four case reports and cumulative review of the literature. *Archives of Surgery*.

[9] Kennedy J, Holt CL, Ricketts RR (1987). The significance of portal vein gas in necrotizing enterocolitis. *American Surgeon*.

[10] Quirke TE (1995). Hepatic-portal venous gas associated with ileus. *American Surgeon*.

[11] Yale CE, Balish E, Wu JP (1974). The bacterial etiology of pneumatosis cystoides intestinalis. *Archives of Surgery*.

[12] Sebastià C, Quiroga S, Espin E, Boyé R, Alvarez-Castells A, Armengol M (2000). Portomesenteric vein gas: pathologic mechanisms, CT findings, and prognosis. *Radiographics*.

[13] Nielsen ST, Olsen A (1985). Gas in the portal venous system. Illustrated by two case reports. *RoFo Fortschritte auf dem Gebiete der Rontgenstrahlen und der Nuklearmedizin*.

[14] Gosink BB (1981). Intrahepatic gas: differential diagnosis. *American Journal of Roentgenology*.

[15] Chezmar JL, Nelson RC, Bernardino ME (1989). Portal venous gas after hepatic transplantation: sonographic detection and clinical significance. *American Journal of Roentgenology*.

[16] Lee C-S, Kuo Y-C, Peng S-M (1993). Sonographic detection of hepatic portal venous gas associated with suppurative cholangitis. *Journal of Clinical Ultrasound*.

[17] Pan HB, Huang JS, Yang TL, Liang HL (2007). Hepatic portal venous gas in ultrasonogram—benign or noxious. *Ultrasound in Medicine and Biology*.

[18] Schulze CG, Blum U, Haag K (1995). Hepatic portal venous gas imaging modalities and clinical significance. *Acta Radiologica*.

[19] Schindera ST, Triller J, Vock P, Hoppe H (2006). Detection of hepatic portal venous gas: its clinical impact and outcome. *Emergency Radiology*.

[20] Gorospe EC (2008). Benign hepatic portal venous gas in a critically ill patient. *The Scientific World Journal*.

[21] Hou SK, Chern CH, How CK, Chen JD, Wang LM, Lee CH (2004). Hepatic portal venous gas: clinical significance of computed tomography findings. *American Journal of Emergency Medicine*.

[22] Monneuse O, Pilleul F, Barth X (2007). Portal venous gas detected on computed tomography in emergency situations: surgery is still necessary. *World Journal of Surgery*.

[23] Radin DR, Rosen RS, Halls JM (1987). Acute gastric dilatation: a rare cause of portal venous gas. *American Journal of Roentgenology*.

[24] Alivizatos G, Skolarikos A, Sopilidis O, Ferakis N, Chorti M (2005). Splenogonadal fusion: report of a case and review of the literature. *International Journal of Urology*.

[25] Chang YS, Wang HP, Huang GT, Wu MS, Lin JT (1999). Sonographic “gastric corona sign”: diagnosis of gastric pneumatosis caused by a penetrating gastric ulcer. *Journal of Clinical Ultrasound*.

[26] Haswell DM, Carsky EW (1979). Hepatic portal venous gas and gastric emphysema with survival. *American Journal of Roentgenology*.

[27] Birnberg FA, Gore RM, Shragg B, Margulis AR (1983). Hepatic portal venous gas: a benign finding in a patient with ulcerative colitis. *Journal of Clinical Gastroenterology*.

[28] Al-Jahdali H, Pon C, Thompson WG, Matzinger FR (1994). Non-fatal portal pyaemia complicating Crohn’s disease of the terminal ileum. *Gut*.

[29] Kirsch M, Bozdech J, Gardner DA (1990). Hepatic portal venous gas: an unusual presentation of Crohn’s disease. *American Journal of Gastroenterology*.

[30] Nguyen HN, Purucker E, Riehl J, Matern S (1998). Hepatic portal venous gas following emergency endoscopic sclerotherapy of gastric varices. *Hepato-Gastroenterology*.

[31] Herman JB, Levine MS, Long WB (1995). Portal venous gas as a complication of ERCP and endoscopic sphincterotomy. *American Journal of Gastroenterology*.

[32] Mognol P, Chosidow D, Marmuse JP (2005). Hepatic portal gas due to gastro-jejunal anastomotic leak after laparoscopic gastric bypass. *Obesity Surgery*.

[33] Oei T, vanSonnenberg E, Shankar S, Morrison PR, Tuncali K, Silverman SG (2005). Radiofrequency ablation of liver tumors: a new cause of benign portal venous gas. *Radiology*.

[34] Stein MG, Crues JV, Hamlin JA (1983). Portal venous air associated with barium enema. *American Journal of Roentgenology*.

